# Impact of Perioperative Proton Pump Inhibitors on Renal Cancer Progression. A Retrospective Study

**DOI:** 10.1002/cam4.71723

**Published:** 2026-03-12

**Authors:** Nicolas Cortes‐Mejia, Ryan Wong, Holger K. Eltzschig, Sergio M. Moreno‐Lopez, Diana Bejarano‐Ramirez, Huang Huang, Juan P. Cata

**Affiliations:** ^1^ Department of Surgery Wyckoff Heights Medical Center New York City New York USA; ^2^ Tilman J. Fertitta Family College of Medicine University of Houston Houston Texas USA; ^3^ Department of Anesthesiology, Critical Care, and Pain Medicine McGovern Medical School UT Health Houston Texas USA; ^4^ School of Medicine Universidad de Los Andes Bogota Colombia; ^5^ Transplant and Hepatobiliary Surgery Department Fundación Santa Fe de Bogotá Bogotá Colombia; ^6^ Epidemiology and Biostatistics Group Graduate School Universidad CES Medellín Colombia; ^7^ Department of Anesthesiology and Perioperative Medicine The University of Texas MD Anderson Cancer Center Houston Texas USA

**Keywords:** ATP4A gene, clear cell renal cell carcinoma, proton pump inhibitors, recurrence, survival

## Abstract

**Background:**

Clear cell renal cell carcinoma (ccRCC) is the most common kidney cancer. Metastatic disease causes most deaths. We investigated the association between ATP4A gene status and survival following nephrectomy. ATP4A encodes H^+^/K^+^ ATPase, targeted by proton pump inhibitors (PPIs). We also examined how perioperative use of these drugs affects cancer progression post‐surgery.

**Methods:**

Using a publicly available database, we employed *in silico* analysis to investigate the association between the *ATP4A* gene and recurrence‐free survival and overall survival. After receiving institutional review board approval from the University of Texas‐MD Anderson Cancer Center (#2020‐0380), we conducted a retrospective analysis to investigate the association between perioperative PPIs use and progression of nonmetastatic ccRCC. We performed a propensity score analysis and utilized inverse probability of treatment weighting to balance potential discrepancies between exposure groups. A Cox proportional hazards model, weighted by inverse probability of treatment weighting, was subsequently fitted for multivariable analysis.

**Results:**

*ATP4A* gene expression analysis showed that high tumor levels were associated with a significant reduction in overall survival. The analysis included a total of 1775 patients with ccRCC. After propensity matching, the analysis revealed that the use of perioperative PPIs was not independently associated with a worse oncological prognosis following nephrectomies.

**Conclusion:**

Elevated ATP4A gene expression correlates with reduced survival, while perioperative PPI use shows no significant impact on survival outcomes.

## 1 Introduction

In 2024, renal cell carcinoma (RCC) was diagnosed in more than 81,610 Americans and is associated with more than 140,000 deaths per year [1, 2, 3]. RCC is the most frequent histological type of kidney cancer, making up over 90% of renal tumors [4]. Improvements in relative survival rates have been reported over the past three decades, although these might be influenced by disease stage migration [3]. Additionally, prognosis varies by stage and subtype, with surgery being the primary treatment approach [4]. The most reliable prognostic factors include tumor size, grade, and the presence of microvascular invasion [5]. RCC biomarkers include clinical markers (IMDC score, neutrophil‐to‐lymphocyte ratio), histologic markers (clear vs. non‐clear cell RCC, sarcomatoid/rhabdoid features), immuno‐oncology markers (PD‐L1, LAG‐3, TIM‐3, TIGIT), genomic markers (TMB, VHL, BAP1, PBR, M1, SETD2 mutations), circulating markers (circulating tumor DNA), transcriptomic markers (ClearCode34, IMmotion gene signatures, BIONIKK classifier), and Carbonic Anhydrase IX (CAIX) as a potential imaging and therapy target [6].

ATP4A is one of two peptide chains that comprise the H^+^/K^+^ ATPase, an enzyme expressed in epithelial cells [7]. The primary function of this protein is to acidify the stomach by exchanging hydrogen ions for potassium ions, maintaining the acidic gastric pH. An *in silico* study identified ATP4A as a potential biomarker for gastric cancer [8]. Another study analyzed gene expression and DNA methylation in gastric cancers and found that *ATP4A* was downregulated in gastric tumor tissues, suggesting that *ATP4A* silencing may contribute to gastric cancer progression [9]. There is scarce evidence about the expression of ATP4A in RCC tumors. However, evidence shows that the protein is expressed and functional in the kidneys of mice and Nile tilapia [10, 11]. While direct studies linking *ATP4A* downregulation to altered tumor acidity and RCC aggressiveness are not published, research indicates that other ion channels and transporters involved in proton extrusion play significant roles in tumor development. For instance, research on papillary renal cell carcinomas suggests that Vacuolar‐type H^+^‐ATPases (V‐ATPase), which regulate the tumor microenvironment through proton extrusion, are involved in tumor progression by promoting extracellular matrix degradation [12].

Proton pump inhibitors (PPIs) block the H^+^/K^+^ ATPase enzyme and exhibit antitumor properties [13, 14, 15, 16, 17, 18]. Various mechanisms of anticancer activity have been suggested, including immunopotentiation, enhanced sensitivity to chemoradiotherapy, induction of apoptosis, suppression of migration, inhibition of detoxification, and inhibition of exosomes carrying microRNA [14, 15, 16, 17, 18]. However, pharmacovigilance research indicates that renal cancer was one of the top five tumor‐associated events in PPI users, with an incidence of 6.02% [19]. Furthermore, PPIs affect the function of key immune effectors, such as natural killer cells, the fecal oral microbiota, and have been shown to reduce the activity of immune checkpoint inhibitors [20, 21, 22, 23, 24]. PPIs are typically administered perioperatively to patients with a history of active peptic ulcers or gastroesophageal reflux disease, and those taking these medications at the time of surgery [25]. It remains unknown whether perioperative PPI administration has any impact on cancer progression after renal cancer surgery. We hypothesize that perioperative PPI use is associated with a diminished risk of clear cell RCC progression and longer recurrence‐free survival (RFS) and overall survival (OS).

## 2 Methods

Initially, we surveyed the publicly accessible database (https://tnmplot.com/analysis/) and compared *ATP4A* expression using RNA sequencing data from normal and ccRCC specimens [26]. We conducted a differential analysis of the *ATP4A* gene across normal, tumor, and metastatic tissues utilizing TNM plot. We employed the Kruskal–Wallis test, followed by Dunn's correction, to compare *ATP4A* expression. Subsequently, we used the Kaplan–Meier Plotter database (https://kmplot.com/analysis/) to assess the relationship between *ATP4A* expression and RFS as well as OS [27]. The database encompasses the GEO, EGA, and TCGA microarray datasets [28]. Specifically, 117 patients were included in the RFS and 530 in the OS analyses. To facilitate visualization in the Kaplan–Meier plots based on *ATP4A* expression, the optimal cutoff value was utilized, and the false discovery rate (FDR) was reported [29]. The Cox‐Mantel (log‐rank) test was used to determine the significance of the findings. The initial analysis was unrestricted with regard to stage, gender, or immune cellularity.

### 2.1 Retrospective Study

This study was conducted after the approval by The University of Texas—MD Anderson Cancer Center Institutional Review Board (#2020–0380), and after obtaining a waiver of informed consent. The study included a retrospective cohort of adult patients who had radical or minimally invasive partial or total nephrectomies for clear cell RCC between June 2016 and November 2023. This study is reported in accordance with the Strengthening the Reporting of Observational Studies in Epidemiology (STROBE) guidelines recommendations [30].

We evaluated adults with confirmed ccRCC. Subjects with other cancer types (i.e., papillary or mixed clear cell/papillary) and benign conditions were excluded, as well as those who had debulking surgeries, patients presenting with metastatic disease, and individuals undergoing emergency nephrectomies. Our primary outcomes measured included RFS and OS. RFS was calculated as the duration in months from the date of surgery to the day of recurrence or last follow‐up [31]. Overall survival was defined as the duration (in months) from the date of surgery to death or last follow‐up [31]. Patients with no recorded recurrence or death were censored at their last follow‐up. Recurrence events were obtained from clinical follow‐up reports. At our institution, recurrences are generally confirmed via imaging and/or biopsy. Patients with indeterminate lesions were not classified as recurrences unless subsequent follow‐up visits led to changes in care.

We collected demographic data (age, sex, body mass index [BMI], ethnicity, and primary race). We also gathered data on ASA physical status classification, oncological data (Fuhrman score and stage), recurrence status (Yes vs. No), mortality (alive vs. deceased), type of resection (partial vs. radical), surgical approach (radical vs. minimally invasive), and duration of anesthesia (minutes).

PPI exposure was defined as evidence of their administration preoperatively (within 30 days of surgery), during surgical admission, and at the first follow‐up visit. Patients who did not take PPIs at any point prior to surgery (within 30 days of the procedure), during the surgical admission, or postoperatively (at follow‐up visits) or intermittently around the perioperative period were regarded as controls.

### 2.2 Statistical Analysis

Demographics and baseline patient characteristics were summarized using descriptive statistics. Categorical variables are shown with absolute counts and percentages, whereas continuous variables are described using either means and standard deviations (SD) or medians and interquartile ranges (IQR), based on the normality assumption assessed by the Shapiro–Wilk test. All analyses were stratified based on preoperative use of PPIs. Given the observational design and potential for indication bias, a propensity score (PS) was estimated through a logistic regression model. This model included covariates with a standardized mean difference (SMD) greater than 0.1 prior to adjustment, clinically relevant factors, and variables associated with PPI exposure according to the literature. The covariates included were: sex, age at surgery, ethnicity, BMI, Charlson Comorbidity Index, ASA classification, tumor stage, Fuhrman grade, surgical procedure, and surgical approach.

Using PS, inverse probability of treatment weighting (IPTW) was applied to balance baseline differences between exposure groups. This approach was chosen over traditional matching or stratification methods due to several advantages. Notably, IPTW retains the entire study population, enhancing statistical power and generalizability, while allowing for the estimation of marginal treatment effects [32]. To mitigate the impact of extreme weights, stabilized weights (SW) were used, defined as the ratio of the marginal probability of treatment to the individual PS‐derived probability. Post‐weighting balance was evaluated using SMDs and overlap plots to ensure adequate comparability between groups.

A doubly robust estimation method was used by adding clinically relevant covariates to the IPTW‐weighted Cox model, ensuring consistent estimates even if either the propensity score model or the outcome model was misspecified [33]. Recurrence‐free survival and OS were analyzed using Kaplan–Meier curves, and multivariable analysis was performed using a Cox proportional hazards model weighted by IPTW. The proportional hazards assumption was verified using Schoenfeld residuals and graphical diagnostics, while collinearity among covariates was assessed with the variance inflation factor. Survival analysis was also conducted according to PPI potency: rabeprazole > esomeprazole > lanzoprazole/dexlanzoprazole > omeprazole > pantoprazole.

A two‐tailed *p*‐value less than 0.05 was considered statistically significant. All statistical analyses and visualizations were performed using Stata 17 (StataCorp. 2021. Stata Statistical Software: Release 17. College Station, TX: StataCorp LLC).

## 3 Results

### 3.1 Association Between *ATP4A* mRNA Expression and RFS and OS in Clear Cell RCC

**FIGURE 1:**
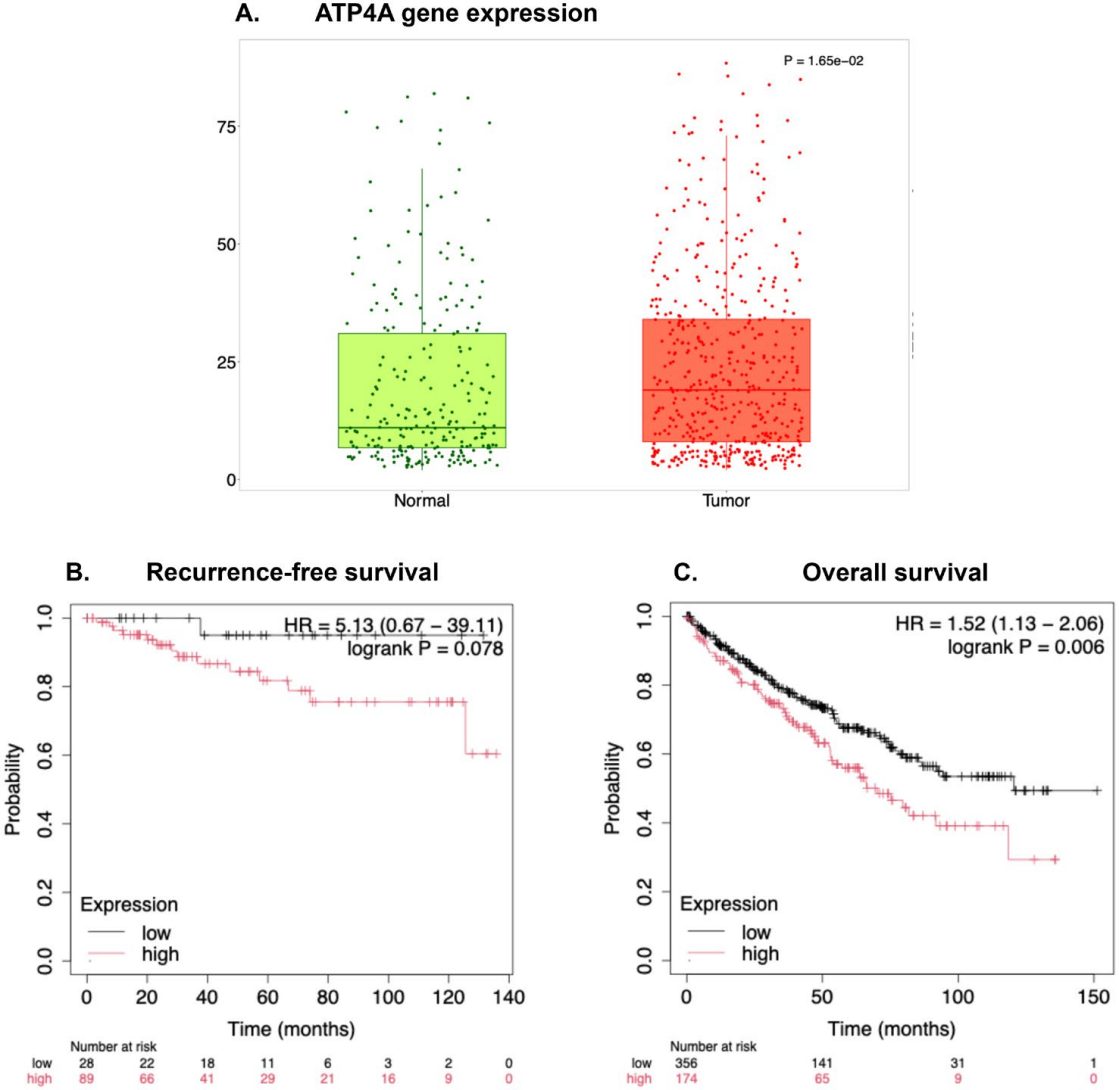
Analysis of ATP4A gene expression and its impact on survival in patients with clear cell renal cell carcinoma (RCC). **(A)** Box plot comparing ATP4A gene expression levels between normal kidney tissues (green) and tumor tissues (red). Expression of ATP4A is significantly upregulated in tumor tissues compared to normal tissues. **(B)** Kaplan–Meier curve for recurrence‐free survival based on ATP4A expression levels in clear cell RCC patients. Patients with high ATP4A expression (red) show a trend toward poorer recurrence‐free survival compared to those with low expression (black), though not statistically significant. **(C)** Kaplan–Meier curve for overall survival based on ATP4A expression levels. High ATP4A expression is significantly associated with reduced overall survival compared to low expression.

*ATP4A* expression data were evaluated in 210 paired tumor and adjacent normal specimens, respectively. As illustrated in Figure 1a, the median [IQR] gene expression was significantly higher in tumors [6, 7, 8, 9, 10, 11, 12, 13, 14, 15, 16, 17, 18, 19, 20, 21, 22, 23, 24, 25, 26, 27, 28, 29, 30, 31, 32, 33, 34, 35, 36, 37, 38, 39, 40, 41] than in normal specimens (11, 6–32, *p* = 0.001). The *ATP4A* levels at the tumor level did not statistically significantly affect RFS (Hazard Ratio, HR = 5.13, 95% CI = 0.67–39.11, cutoff = 0, FDR = 100%, *p* = 0.078, Figure 1b). However, increased expression of the gene at the tumor level was significantly associated with shorter OS (HR = 1.52, 95% CI = 1.13–2.06, cutoff = 1, FDR = 50%, *p* < 0.0004, Figure 1c).

### 3.2 Association Between PPI Use, ccRCC Recurrence, and Survival

**FIGURE 2:**
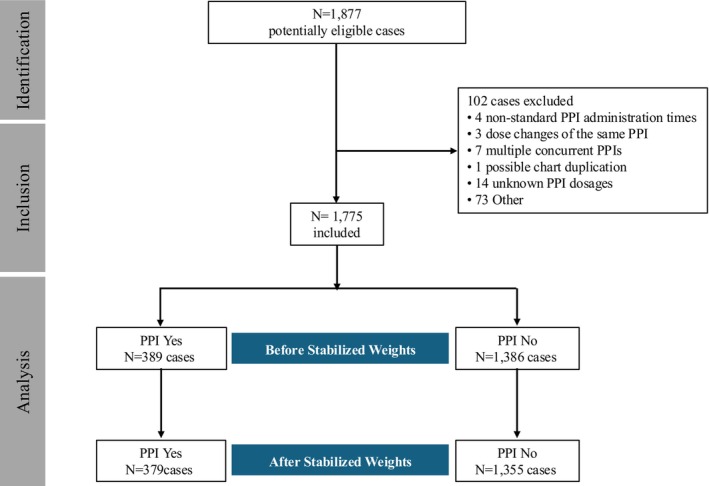
The diagram outlines the number of patients identified, screened, assessed for eligibility, and included in the analysis.

Our retrospective cohort comprised 1775 patients with ccRCC (see Figure 2). Among these, 389 patients were receiving PPIs perioperatively. As illustrated in Table 1, patients in the PPI group were statistically significantly older (median, interquartile range (IQR): 62, 54–69 years versus 60, 50–67 years; *p* < 0.001), had a higher proportion of females (40.1% versus 34.1%; *p* = 0.026), and were less likely to be Hispanic or Latino (79.9% versus 70.6%; *p* < 0.001) compared to the control group. Eleven patients (2.8%) in the PPI cohort had a history of receiving immunotherapy (nivolumab, *n* = 8; pembrolizumab, *n* = 2, and ipilumab, *n* = 1), while 34 (2.4%) in the non‐PPI cohort received immunotherapy (nivolumab, *n* = 30; pembrolizumab, *n* = 2; ipilumab, *n* = ; and cemiplimab = 1). Analysis of missing values showed a loss of 2.03% in the ethnic origin variable and 0.28% in the Fuhrman score. Given the low proportion of missing values, no imputation of these variables was performed, and it was decided to exclude participants with missing values from the analysis.

**TABLE 1:** Demographic, tumor, and perioperative data.

Characteristics	Proton pump inhibitor use		
	NO (*N* = 1386)	YES (*N* = 389)	*p*
Age (years)	60 (50–67)	62 (54–69)	< 0.001
Sex			0.029
Male	913 (65.87)	233 (59.9)	
Female	473 (34.13)	156 (40.1)	
BMI (kg/m2)	30.92 (27.1–35.5)	31 (27.5–35.6)	0.96
ASA physical status			0.56
ASA 1–2	205 (14.79)	53 (13.62)	
ASA 3–4	1181 (85.21)	336 (86.38)	
Charlson comorbidity index	4 (3–4)	4 (3–5)	< 0.001
Ethnicity			0.001
Hispanic or Latino	377 (27.2)	72 (18.51)	
Not Hispanic or Latino	979 (70.63)	311 (79.95)	
Unknown	30 (2.16)	6 (1.54)	
Final procedure			0.70
Partial nephrectomy	822 (59.31)	235 (60.41)	
Radical nephrectomy	564 (40.69)	154 (39.59)	
Final approach			0.87
MIS	756 (54.55)	214 (55.01)	
Open	630 (45.45)	175 (44.99)	
Stage			0.69
I	810 (58.44)	235 (60.41)	
II	28 (2.02)	5 (1.29)	
III	524 (37.81)	141 (36.25)	
IV	24 (1.73)	8 (2.06)	
Fuhrman score	2 (2–3)	3 (2–3)	0.23
Anesthesia time (min)	227 (159–280)	226 (153–280)	0.57
Procedure duration (min)	153 (97–206)	150 (92–210)	0.82

*Note:* Data are presented as median (IQR) for continuous measures, and *n* (%) for categorical measures.

Abbreviations: ASA, American Society of Anesthesiologists; BMI, body mass index; min, minutes; MIS, minimally invasive surgery.

Table 2 also illustrates significant imbalances in sex, age, ethnicity, and the Charlson comorbidity index before IPTW implementation. Before IPTW, the overall incidence recurrence rate was 5.11 events per 100 person‐years of follow‐up (95% CI: 4.50 to 5.81), and the mortality rate was 2.69 deaths per 100 person‐years of follow‐up (95% CI: 2.25 to 3.21). The rate of recurrence was 11.83% (*n* = 46) and 13.56% (*n* = 188, *p* = 0.37) for patients taking and not taking PPIs, respectively.

**TABLE 2:** SW analysis between patients taking and not taking PPIs.

Characteristics	Before Stabilized Weights					After Stabilized Weights				
	PPI NO		PPI YES		SMD	PPI NO		PPI YES		SMD
	(*N* = 1386)		Yes (*N* = 389)			(*N* = 1355)		(*N* = 379)		
	Mean	SD	Mean	SD		Mean	SD	Mean	SD	
Sex	0.40	0.49	0.34	0.47	0.12	0.35	0.48	0.35	0.48	−0.01
Age	61.22	10.91	58.44	12.22	0.24	59.29	11.38	59.07	12.10	0.02
Ethnicity	0.812	0.392	0.72	0.45	0.21	0.75	0.43	0.74	0.44	0.02
BMI	32,035	6.546	32.07	6.84	0.00	32.06	6.77	32.05	6.86	0.00
CCI	3.838	1.221	3.63	1.29	0.16	3.70	1.26	3.68	1.29	0.01
ASA PS	0.866	0.341	0.85	0.35	0.04	0.86	0.35	0.86	0.35	0.01
Stage	0.382	0.487	0.40	0.49	−0.04	0.39	0.49	0.40	0.49	−0.01
Fuhrman score	2.605	0.738	2.56	0.70	0.06	2.56	0.74	2.57	0.70	−0.01
Final approach	0.455	0.499	0.45	0.50	0.00	0.46	0.50	0.46	0.50	0.01
Final procedure	0.398	0.490	0.41	0.49	−0.02	0.41	0.49	0.41	0.49	0.00

Abbreviations: ASA PS, American Society of Anesthesiologists Physical Status; BMI, body mass index; CCI, charlson comorbidity index.

**FIGURE 3:**
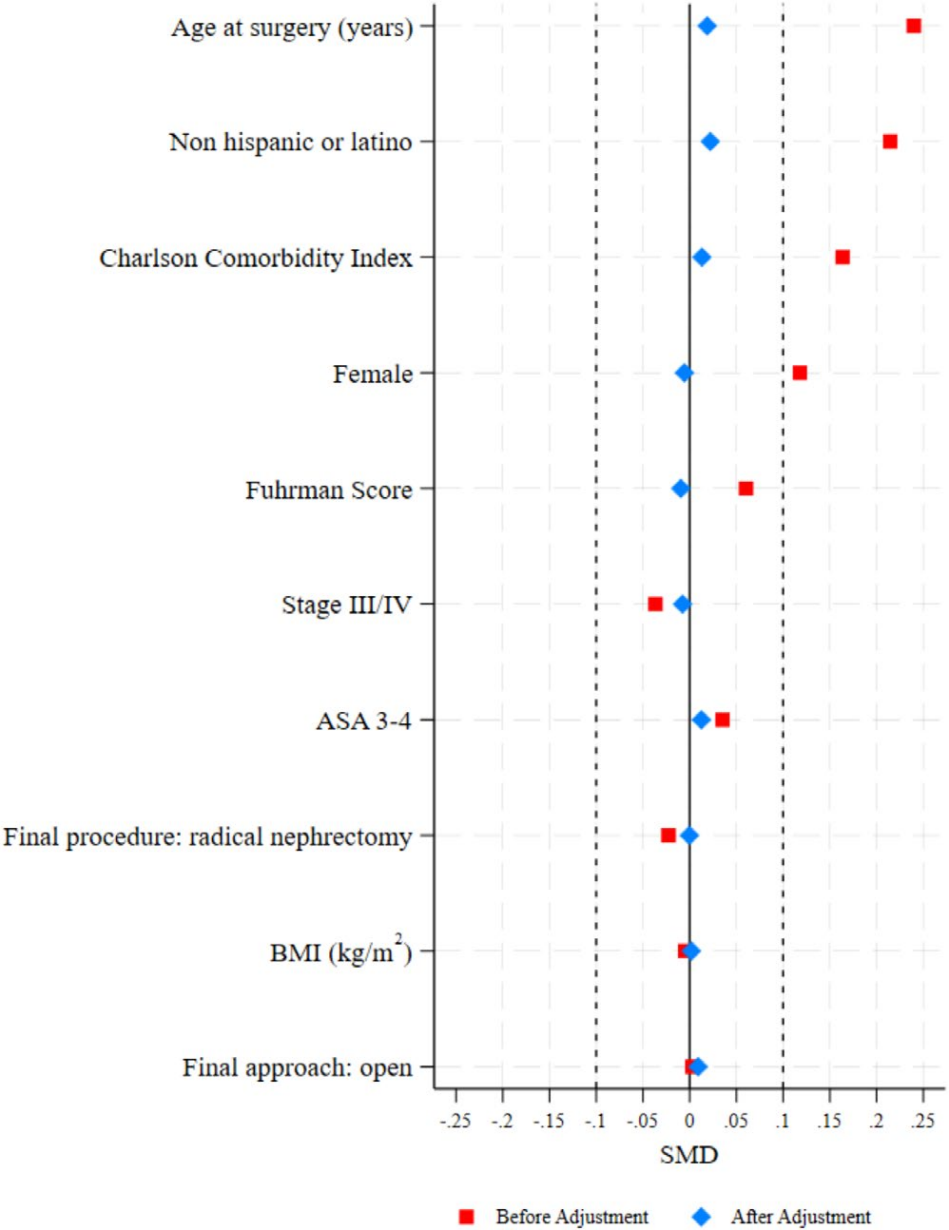
The figure presents the standardized differences for key covariates before and after inverse probability weighting between the two comparison groups. Each covariate is plotted along the y‐axis, and the standardized difference is represented on the x‐axis.

**FIGURE 4:**
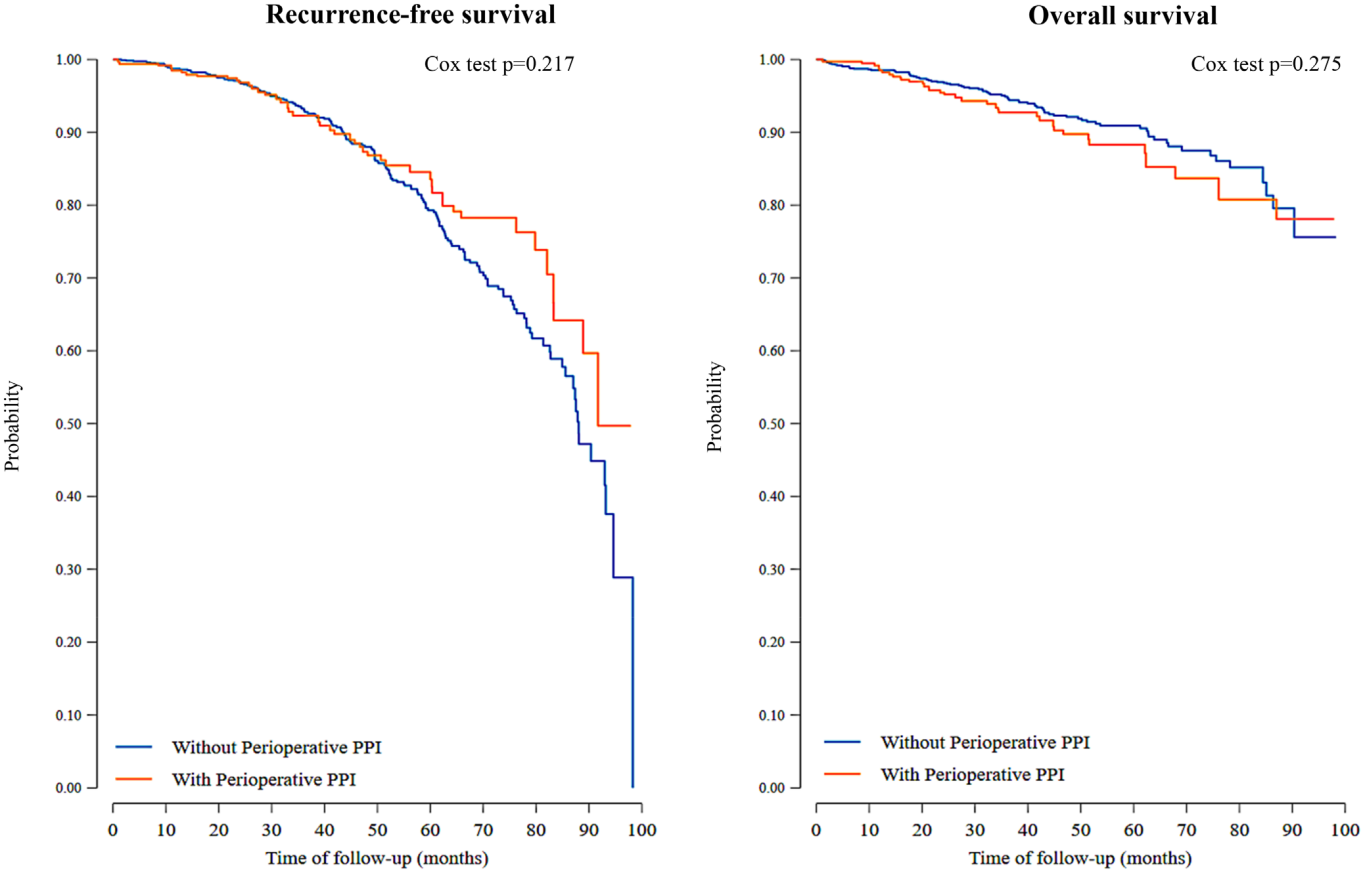
Kaplan–Meier survival curves comparing outcomes between patients who received perioperative proton pump inhibitors (PPI) and those who did not. Left panel: Recurrence‐free survival over time (days). The orange line represents patients treated with perioperative PPI, while the blue line represents patients without PPI. No statistically significant difference was observed between groups (Cox test *p* = 0.217). Right panel: Overall survival over time (days), comparing the same patient groups. Again, no statistically significant difference was detected (Cox test *p* = 0.275). Both graphs suggest that perioperative PPI use was not associated with significant differences in recurrence‐free or overall survival outcomes.

After IPTW, the standard mean difference between imbalanced variables was substantially reduced, indicating good balance between groups (Table 2 and Figure 3). After IPTW, 379 and 1355 patients remained in the PPIs group and control group, respectively. The unadjusted univariate log‐rank estimates revealed no statistically significant differences in RFS (HR = 0.82, 95% CI = 0.58–1.16, *p* = 0.263) and OS (HR = 1.19, 95% CI = 0.79–1.79, *p* = 0.275) between groups (Figure 3). After IPTW, the incidence recurrence rate was 5.09 events per 100 person‐years of follow‐up (95% CI: 4.46 to 5.84), and the mortality rate was 2.2 deaths per 100 person‐years of follow‐up (95% CI: 1.87 to 2.67). After accounting for gender, BMI, age, ethnicity, CCI, ASA physical status, tumor stage, Fuhrman score, procedure, and final surgical approach, the multivariate Cox proportional hazards analysis revealed no statistically significant association between the perioperative PPIs use and RFS (HR = 0.79; 95% CI = 0.55–1.14, *p* = 0.214) or OS (HR = 1.2; 95% CI = 0.72–1.84, *p* = 0.405, Figure 4) (Table 3). With regard to model diagnostics, goodness‐of‐fit tests based on the proportional hazards assumption were performed, demonstrating that this assumption was satisfied. No issues of collinearity were detected. An assessment of the model's fit utilizing Cox‐Snell residuals indicated that the model appropriately accommodated the data.

**TABLE 3:** Multivariate analysis for recurrence‐free and overall survival.

Variables	Recurrence‐free			Overall Survival		
	HR	95% CI	P value	HR	95% CI	P value
Perioperative PPI						
No	1	(ref)		1	(ref)	
Yes	0.796	0.556–1.14	0.214	1,2	0,782‐1,84	0,405
Sex						
Male	1	(ref)		1	(ref)	
Female	0.927	0.686–1.25	0.621	1,06	0,725‐1,56	0,753
Age (years)	1.01	0.98–1.04	0.56	1	0,968‐1,04	0,954
Ethnicity						
Hispanic or Latino	1	(ref)		1	(ref)	
Not Hispanic or Latino	1.28	0.889–1.83	0.185	1,3	0,784‐2,16	0,31
Body mass index	0.998	0.978–1.02	0.839	1,03	1–1,06	0,047
Charlson comorbidity index	0.937	0.717–1.23	0.637	1,5	1,14‐1,96	0,004
ASA physical status						
1–2	1	(ref)		1	(ref)	
3–4	1.02	0.679–1.53	0.928	1,03	0,603‐1,76	0,911
Stage						
I/II	1	(ref)		1	(ref)	
III/IV	2.63	1.69–4.08	0.001	2,15	1,27‐3,62	0,004
Fuhrman score	2.71	2.17–3.4	0.001	1,82	1,34‐2,46	0,001
Final surgical approach						
Minimally invasive	1	(ref)		1	(ref)	
Open	1.82	1.37–2.41	0.001	1,93	1,29‐2,88	0,001
Final surgical procedure						
Partial nephrectomy	1	(ref)		1	(ref)	
Radical nephrectomy	1.66	1.07–2.58	0.023	1,36	0,8‐2,3	0,258

Abbreviation: ASA: American Society of Anesthesiologists.

Lastly, we tested the association between different PPIs with RFS based on their potency. The analysis showed no statistically significant association (Figure S1).

## 4 Discussion

Our gene analysis demonstrates that high *ATPA4A* gene expression in ccRCC specimens is significantly associated with reduced OS and has a marginally negative effect on RFS. To the best of our knowledge, no prior studies have evaluated the influence of *ATPA4A* gene expression on the progression of clear cell RCC. However, *ATP4A* has been investigated as a prognostic biomarker for premalignant lesions, as well as gastric and esophageal carcinomas [9, 34, 35, 36, 37]. Akhtar et al. showed that high expression of *ATP4A* was linked to decreased OS in gastric cancers [34].

Our research also demonstrated that the administration of PPIs prior to, during, and following surgical procedures was not associated with statistically significant alterations in RFS or OS. The effect of perioperative PPI administration on cancer progression has not been explored in prior RCC studies. However, Ho et al. investigated the association between long‐term PPI use and recurrence after liver resections for hepatocellular carcinoma. The authors found that PPI users had a 50% lower risk of HCC recurrence than the non‐PPI group after hepatectomies [38]. Our findings may have several explanations. We limited the duration of exposure to PPIs to the perioperative period. Zhang et al. reported that PPI use was associated with an increased incidence of renal cancer [19]. However, the duration of exposure to PPIs in Zang's study was not specified. A study conducted by Kwon et al. revealed that a history of PPI use is associated with a 19% increased risk of lung cancer [39]. However, the study also indicated that prolonged exposure (> 30 days) to PPIs reduced the risk of developing new lung cancer, but it increased the mortality risk. A Swedish study including a 754,118 PPI users (> 180 days of cumulative use) showed a 10% increased risk of developing colorectal carcinoma compared to the general population (1.10, 95% CI = 1.06 to 1.13) for both sexes [40]. Hence, it is plausible to hypothesize that a prolonged duration of exposure might have impacted survival outcomes within our patient cohort. However, receptor blockade with PPIs may have failed to produce direct cellular effects in cancer cells and positively impact survival because ATPA4A expression may have been low, heterogenous, uncoupled, or biologically irrelevant compared with dominant oncogenic pathways (i.e., mutations in AKT/mTOR signaling) in our patient population [41, 42].

Our work did not explore the association between perioperative PPI use and disease progression in patients with metastatic RCCs. It is worth noting that coadministering PPIs with nivolumab has been associated with shorter survival in patients with metastatic renal cancer [43]. However, this contradicts the findings of a retrospective study using data from the GETUG‐AFU 26 NIVOREN Phase II study in which patients received nivolumab [44]. It should be noted that, at our institution, a small fraction (2.5%) of patients with RCCs are routinely prescribed pembrolizumab or other immunotherapies. This limited the statistical power to assess the association between PPI and survival, with or without immunotherapies. Del Re et al. investigated the effect of coadministering PPIs and vascular endothelial growth factor receptor tyrosine kinase inhibitors. The authors found an association between PPIs and shorter progression‐free survival in metastatic renal cancer patients treated with pazopanib and cabozantinib [45].

In the past two decades, there has been an increased enthusiasm for finding perioperative interventions that could modify cancer recurrence, including in patients with ccRCC. While a retrospective study suggested an association between epidural anesthesia (in comparison to opioid‐based analgesia) and better survival outcomes in patients with renal carcinoma, randomized controlled trials have shown no oncological benefits with the use of regional anesthesia in several different populations [46, 47]. Similarly, an investigation that included patients with urological malignancies who were allocated to receive propofol‐based total intravenous versus volatile anesthesia demonstrated no differences in survival [48]. Lastly, a previous investigation by our group found that intraoperative infusion of dexmedetomidine was not associated with clinically meaningful changes in RFS or OS [31].

Our study has limitations. First, we have limited information on the duration of exposure prior to surgery, and we only investigated the association with their use in the first two to four weeks after surgery. Additionally, the potential for unmeasured (i.e., smoking status), residual, and unadjusted confounding by indication cannot be excluded. Patients with greater comorbidity may have been more frequently prescribed PPIs, potentially biasing mortality estimates. Our cohort comprises data from a single cancer center, thus our findings may not be applicable to a community setting. Second, we did not conduct a dose‐dependent analysis because we lacked reliable information on the dose of PPIs for all patients prior to surgery [19]. Third, we did not investigate the interaction with other drugs given perioperatively (i.e., antimicrobials) or the oral microbiota, which may have either mitigated or masked the effects of PPIs [49]. A recent study found that PPI use was associated with a decrease in Shannon's diversity and this may have impacted response to ICIs [50, 51]. Fourth, we did not investigate the association of each individual PPI drug. In a prior study, esomeprazole was associated with the highest proportion (36%) of PPI‐related cancers compared to rabeprazole (1.7%) [19]. Fifth, our PPI exposure definition may not have captured the biologically relevant window of PPI influence on tumor biology or the immune microenvironment, nor the interaction with drugs such as immunotherapy agents. Lastly, the *ATP4A* gene encodes for the H^+^, K^+^‐ high molecular weight catalytic alpha subunit but not the smaller, heavily glycosylated beta subunit. Future work from our group is dedicated to investigating the association between *ATP4B* and its prognosis in RCC.

In conclusion, clear cell RCCs showed higher expression of the *ATP4A* gene compared to their normal tissue counterparts. A high expression of the *ATP4A* gene in clear cell RCC tumors was associated with shorter survival. The perioperative administration of proton pump inhibitors did not have a statistically significant impact on survival outcomes.

## Author Contributions

**Nicolas Cortes‐Mejia:** data curation (equal), writing – original draft (equal). **Ryan Wong:** data curation (equal). **Holger K. Eltzschig:** methodology (equal), supervision (equal), writing – review and editing (equal). **Sergio M. Moreno‐Lopez:** formal analysis (equal), writing – original draft (equal), writing – review and editing (equal). **Diana Bejarano‐Ramirez:** formal analysis (equal), methodology (equal), writing – original draft (equal), writing – review and editing (equal). **Huang Huang:** conceptualization (equal), writing – original draft (equal). **Juan P. Cata:** conceptualization (equal), investigation (equal), methodology (equal), project administration (equal), writing – original draft (equal), writing – review and editing (equal).

## Funding

The authors have nothing to report.

## Conflicts of Interest

The authors declare no conflicts of interest.

## Supporting information


**Figure S1:** The figure illustrates the impact of PPI potency (rabeprazole > esomeprazole > lanzoprazole/dexlanzoprazole > omeprazole > pantoprazole) on recurrence‐free survival.

## Data Availability

Data might be shared upon reasonable quest and approval by the corresponding author.
