# Supplementary material for: Impact of Perioperative Proton Pump Inhibitors on Renal Cancer Progression. A Retrospective Study
Source: Cancer Med. 2026 Mar 12;15(3):e71723. doi: 10.1002/cam4.71723 (PMC13093733; doi:10.1002/cam4.71723)
Supplement: Supplementary file 1 — Figure S1: The figure illustrates the impact of PPI potency (rabeprazole > esomeprazole > lanzoprazole/dexlanzoprazole > omeprazole > pantoprazole) on recurrence‐free survival. [file CAM4-15-e71723-s001.docx]

**Supplementary figure 1.** Impact of PPI use on recurrence-free survival

Cox test p=0.303


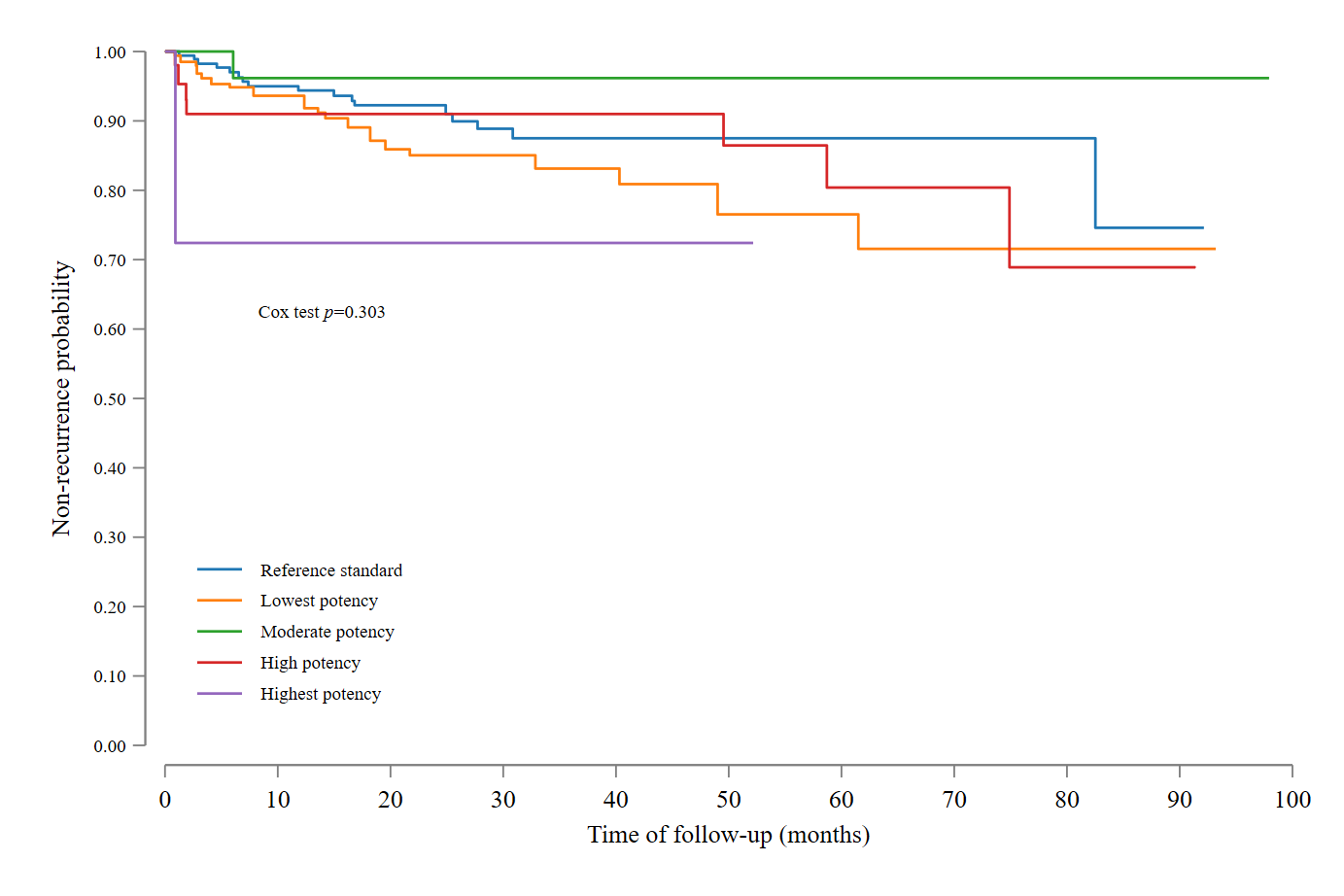


Probability

**Recurrence-free survival**


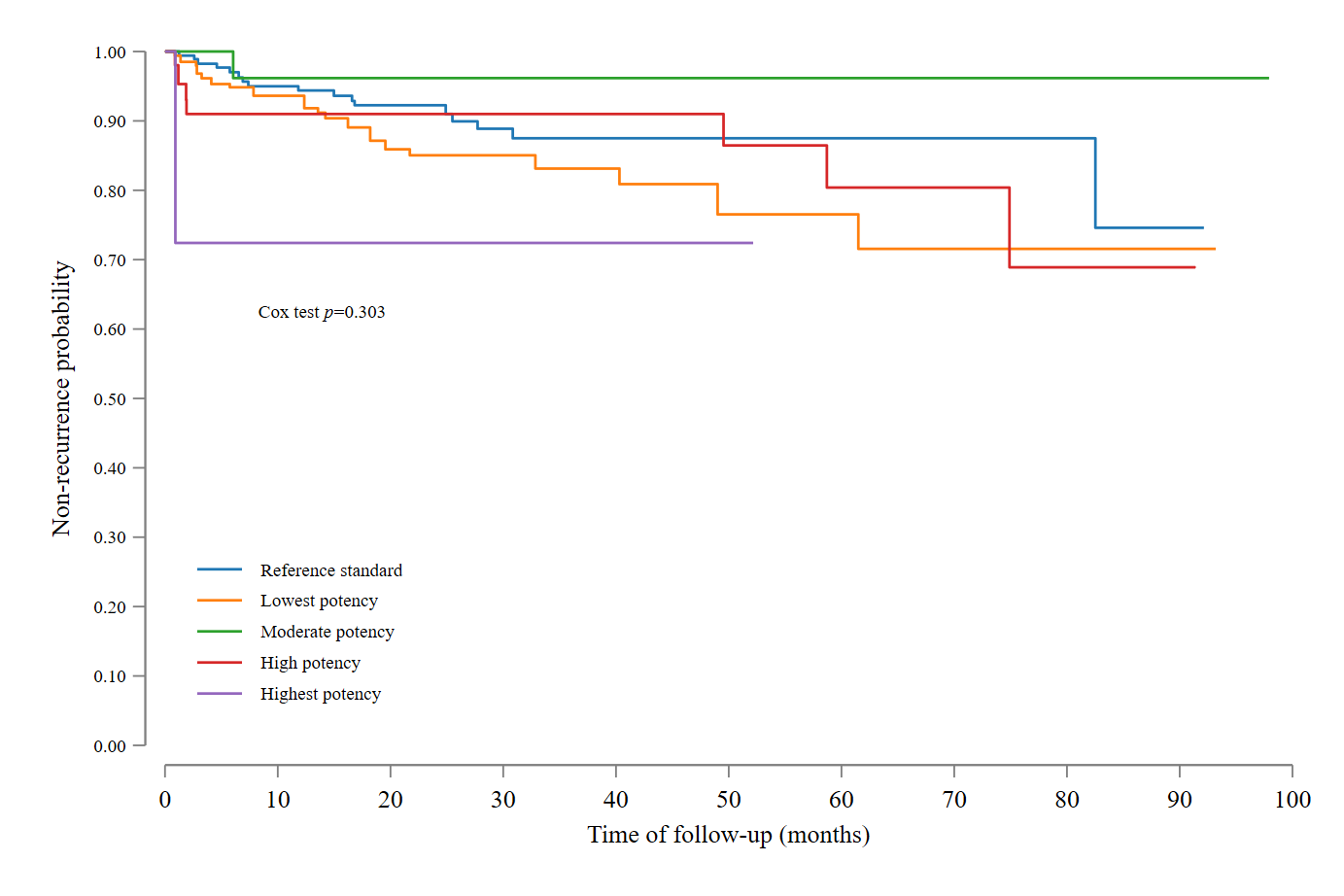


Omeprazolele

Pantoprazole

Rabeprazole

Esomeprazole

Lanzoprazole/dexlanzoprazole

Cox test p=0.303
